# Exploring the factors influencing college students’ learning satisfaction in generative AI-supported MOOCs learning environment: a learning experience framework perspective

**DOI:** 10.3389/fpsyg.2025.1633686

**Published:** 2025-10-27

**Authors:** Peipei Lv, Xin Wang, Yong Nie, Ruixin Wu, Lu Wang

**Affiliations:** ^1^Faculty of Education, Shaanxi Normal University, Xi’an, China; ^2^Teaching Center, Guangzhou Open University, Guangzhou, China

**Keywords:** GenAI, MOOCs learning environment, learning experience, learning satisfaction, college students

## Abstract

**Introduction:**

Massive Open Online Courses (MOOCs) are gradually integrating GenAI technologies to provide personalized support. However, the mechanisms underlying the factors influencing learners’ satisfaction in GenAI-supported MOOCs remain unclear. Under learning experience perspective, this study constructs a model of factors influencing college students’ learning satisfaction (LS) that includes four dimensions: GenAI-supported MOOC learning environment (LE), teacher-student interaction (TSI), student–student interaction (SSI) and learning outcomes (LO).

**Method:**

Data from 402 college students’ questionnaires were collected in GenAI-supported MOOC courses, which was analyzed by IBM SPSS 25 and AMOS 26 software platforms. A structural equation model (SEM) was used to validate the theoretical model of satisfaction influencing factors.

**Results and discussion:**

Results found that: (1) students’ overall LS in GenAI-supported MOOCs is high, indicating the environment can satisfy students’ most learning needs; (2) although LE does not directly affect LS, it positively influences LS through the mediation of LO, which suggests that learners’ perception of LE needs to be translated into the actual LO before it can improve LS; (3) TSI has a significant positive impact on LO, but a negative impact on satisfaction, indicating that GenAI intervention may lead to emotional detachment or excessive expectations; (4) SSI promotes LO, but does not have a significant impact on LS, reflecting that the value of peer collaboration has not been fully embodied in GenAI environment. In GenAI-supported MOOCs, improving teachers’ GenAI collaboration ability, balancing human-computer roles, and strengthening emotional support are the future directions to enhance LS. This study provides empirical evidence for the in-depth application and effect enhancement of GenAI in MOOCs.

## 1 Introduction

Characterized by massive, open and online learning, MOOCs play an important role in expanding access to higher education and facilitating student learning (Kumar and Kumar, 2020). Strong interest and publicity for MOOCs has led almost every university to offer at least one such course (Hew et al., 2020). Shah reports that by the end of 2018, more than 900 universities around the world offered 11,400 MOOCs with a total enrollment of 101 million learners (Shah, 2018), and in the past few years the number of courses and learners have also been increasing rapidly and continuously (M. Zhu et al., 2018). However, it has been found that learner satisfaction in massively developed online courses tends to be lower than in traditional offline classrooms (Pickering and Swinnerton, 2019), and in particular, inappropriate assessment, lack of support, and inappropriate teacher-student interactions can lead to lower learner satisfaction, which in turn affects student learning outcomes (Nguyen, 2022).

In recent years, the rapid development of Generative Artificial Intelligence (GenAI) technology has brought new opportunities for MOOCs teaching. GenAI is a technology based on algorithms and models to generate content such as text, images, sound, video, code, etc., which is characterized by content originality, multimodal capabilities, etc.(Bommasani et al., 2022). GenAI is able to automatically generate content according to human instructions, which has a direct impact on the educational process directly (Wei Fei and Zhiting, 2025). Research has shown that large-scale language models, such as ChatGPT, can act as virtual tutors to answer students’ questions and provide personalized guidance (Rachh and Kavatagi, 2024) and create more productive learning environments (Kasneci et al., 2023). GenAI-supported MOOCs learning environments have the ability to implement learning analytics, recommender systems, adaptive learning, intelligent assessment, and more, can provide students with a richer learning experience (Anderson et al., 2025). For example, the international online course platform Coursera has integrated GenAI large models to introduce features such as AI-assisted grading and AI-based Viva exams (SME, 2024). Meanwhile, China’s online course platform MOOC.cn has embedded Deepseek’s GenAI “Xiaomu,” enabling functions like AI-generated questions and AI-curated learning paths (Jun, 2025).

Although the integration of GenAI technology into MOOCs has been steadily advancing, existing research primarily focuses on utilizing AI within MOOCs for behavior predicting and cognition analyzing (Chonraksuk and Boonlue, 2024; Dai et al., 2025), fostering critical thinking (Wright and Publications, 2025), and other related areas, empirical research evaluating the effectiveness and impact mechanisms of GenAI-supported MOOC learning environments from the learner’s perspective remains relatively limited (Pang and Wei, 2025). Unlike objective measures focused on behavior or cognition, learning satisfaction serves as a subjective indicator for assessing the learning process and outcomes. It more directly reflects learners’ experiences and perceptions within GenAI-supported learning environments, revealing the psychological processes at play in complex human-computer interactions. This provides a crucial complement to comprehensively evaluating the learning effectiveness of GenAI-supported MOOCs (Banihashem et al., 2023; Belkina et al., 2025; Chu, 2025; Sun et al., 2008; Wu et al., 2010; Zhang et al., 2024). Therefore, investigating student learning satisfaction and its influencing factors in GenAI-supported MOOCs learning environments is a crucial step in enhancing the quality of such learning environments, attracting widespread attention from researchers (Sharif Nia et al., 2023).

Currently, research on the factors influencing satisfaction with GenAI-supported MOOCs learning is limited in perspective, focusing mostly on assessing students’ experience and outcomes when using a specific feature of GenAI in the course, such as writing assistance, quick answers to questions, etc (van Niekerk et al., 2025). However, distributed cognition theory emphasizes that cognition does not occur in isolation. Instead, it is distributed across interactions among learners, learning environments, and cultures, forming a multi-system that encompasses all entities involved in cognitive activities—including cognitive agents and cognitive environments (Bosch et al., 2019). In GenAI-supported learning, we cannot evaluate learners’ cognitive engagement and satisfaction solely based on the utility and ease of use of technological tools. Instead, we must embed these tools within the broader learning ecosystem—encompassing environment, interpersonal dynamics, and cognition—for a holistic assessment (Teräs, 2022). The learning experience, as the source of learners’ subjective perceptions and satisfaction with their participation, involves evaluating multiple dimensions including cognition, behavior, and emotion (Li et al., 2023). Therefore, it is important to study the influencing factors of satisfaction with GenAI-supported MOOC learning from the perspective of students’ learning experience (Anderson et al., 2025). This study focuses on college students’ experiences of the learning environment, interactions (teacher-student interaction, student–student interaction), learning outcomes and their impact on learning satisfaction when using GenAI tools to support MOOCs learning. From learning experience perspective, this study constructs a hypothetical model of factors influencing college students’ MOOC learning satisfaction supported by GenAI. Structural equation modeling is employed to validate the model’s fit, aiming to meet learners’ educational needs, enhance the quality and effectiveness of GenAI-supported learning, and provide a theoretical foundation for personalized learning and lifelong learning in the intelligent era.

## 2 Conceptual framework

### 2.1 Online learning experience

“Learning experience” refers to the subjective feelings and overall experience that learners experience in a particular learning environment. The Glossary of Education Reform in the United States (The Glossary of Education Reform) defines the learning experience as the synthesis of any interaction, curriculum, environment, and other factors that produce an experience during the learning process (Bruso et al., 2020). Learners incorporate their unique personality traits and preferences into the learning environment, and these factors influence their effective use of skills and strategies to achieve academic goals. Scholars have different interpretations of the learning experience in online learning environments (shown in Table 1). Udo et al. (2011) argue that the online learning experience consists of four aspects: course platform, teacher-student interaction, student–student interaction, and individual learning; Wilson et al. (1997) argue that the course learning experience consists of clear goals, good instruction, appropriate load, appropriate assessment, and emphasis on independence are composed of five aspects; Liu et al. (2016) believe that the online course learning experience consists of four aspects: the course environment experience, the learning activity experience, and the perception and evaluation of learning effects; Paechter et al. (2010) believe that the online learning experience consists of the course learning materials and the online course environment, teacher-student interaction, student–student interaction, individual learning process and learning outcomes. In general, the concept of “online learning experience” has gone through an evolutionary process from technology-oriented to learner experience-centered, emphasizing the enhancement of subjective experience in the learning process in all aspects.

**Table 1:** The composition dimensions of the online learning experience.

Learning experience	Constitutional dimension	Literature
Online learning experience	Course platform, Teacher-Student interaction, Student–Student interaction, Individual learning	Udo et al. (2011)
Course learning experience	Clear goals, Good teaching, Appropriate burden, Proper evaluation, and Emphasis on independence	Wilson et al. (1997)
Online course learning experience	Course environment experience, Learning activity experience, and Perception of learning outcomes	Liu et al. (2016)
Online learning experience	Course design, Learning materials and Online course environment, Teacher-student interaction and Student–student interaction, learning process and Learning outcomes	Paechter et al. (2010)

### 2.2 GenAI-supported MOOC learning experience

GenAI technologies offer new paths to optimize the MOOCs learning experience (Ouyang and Jiao, 2021). On the one hand, GenAI can participate in the teaching and learning process as intelligent tutors and learning partners (Rachh and Kavatagi, 2024). For example, embedding AI learning assistants in MOOCs platforms can answer students’ questions and provide personalized advice in real time (Yılmaz and Yılmaz, 2022). This enhances the interactivity of online learning (Kasneci et al., 2023) and helps to alleviate the lack of support due to the imbalanced teacher-student ratio in traditional MOOCs. On the other hand, GenAI is able to recommend customized learning resources and exercises based on learners’ behavioral data and cognitive levels, enabling adaptive learning paths. In summary, generative AI plays multiple roles, such as “virtual tutor” and “intelligent assistant teacher,” to enhance the online learning experience in terms of cognitive support, interactive support and personality support. However, there are some challenges in GenAI-supported MOOCs learning. For example, in terms of emotional experience, Rudolph et al. found that learners were ambivalent about using GenAI, relying on its efficiency but also experiencing anxiety due to a sense of “cheating” (Rudolph and Tan, 2023).

GenAI-supported MOOCs learning is a complex learning environment (Gutiérrez-Páez et al., 2023; Lang et al., 2025). The different interaction interfaces of GenAI create differentiated learning environments, and the different performances of the instructor and the learning peers during the process of using GenAI are all important components of the learning experience. In this study, we refer to Paechter et al.’s dimensions of online learning experience, and define GenAI-supported online learning experience as the comprehensive perception of the learning environment, interactions (teacher-student interactions, student–student interactions), and learning outcomes during the process of using a GenAI tool to support learning.

### 2.3 Learning satisfaction and influencing factors

Learning satisfaction is an important indicator of learning effectiveness, and is the learner’s evaluative opinion and felt experience of the quality of the learning service in which they are engaged, which is formed by a rational and emotional comparison of the actual learning experience and expectations (Du, 2022). High satisfaction often implies that the learning experience is enjoyable and needs are met, which can have a positive impact on promoting continued willingness to learn and learning outcomes. In recent years, several studies have been devoted to identifying the key influencing factors of online learning satisfaction and developing corresponding theoretical models. GenAI-supported studies on MOOC learning satisfaction have mainly focused on the study of the relationship between motivation to use and the factors influencing satisfaction, and between learning outcomes and satisfaction. Kim et al. compared the perceptions of instructors and students toward GenAI and found that the overall attitudes of students toward the use of GenAI were higher than those of instructors. However, both perceived GenAI as having more negative than positive effects on learning (Kim et al., 2025a). In addition, performance expectations and effort expectations significantly influence students’ willingness to integrate ChatGPT into their learning practices (Khlaif et al., 2024). The learning experience in the GenAI-supported MOOC learning environment for undergraduates in this study includes learning environment, interaction and outcome aspects. The learning experience was used to construct a model of GenAI-supported MOOC learning satisfaction for college students, which provides a reference for GenAI to help improve the quality of education and teaching.

## 3 Hypothesis development

Based on the above literature review and theoretical framework, we focus on GenAI-supported MOOCs environment from the perspective of learning experience and distill four key factors affecting college students’ learning satisfaction, including learning environment, teacher-student interaction, student–student interaction, and learning outcomes.

Learning environment is the foundation on which learning takes place, including the technological and resource environments (Mi and Wu, 2017). Deci and Ryan (1985) suggest that the ideal external environment is supportive and meets an individual’s psychological needs, thereby promoting positive psychological development and high-quality learning outcomes. Traditional educational environments often fail to stimulate curiosity, resulting in limited learning interactions. GenAI creates an environment that is conducive to fostering curiosity, helping to create a strong connection between instructor and student, and fostering the learner’s desire to learn based on knowledge (Evans et al., 2023). In online learning, GenAI enables output to provide personalized learning resources based on students’ different styles of input, thus promoting group and peer-to-peer interaction, learning engagement, and effectiveness (Lu and Ba, 2025).

GenAI reinvents personalized education by introducing interactive and engaging learning features that ignite learner interest and engagement (Mittal et al., 2024). In terms of learning support, varying the level of difficulty to provide challenges or basic exercises based on the performance of different students, and providing feedback on tailored resources and suggestions after assessment, GenAI is an efficient learning sup-port tool, although it falls short at the graduate level, but excels in knowledge retrieval (Lang et al., 2025; Simon Frieder et al., 2023).

*H1*: Learning environment has a direct and significant effect on teacher-student interaction.

*H2*: Learning environment has a direct and significant effect on student-student interaction.

*H3*: Learning environment has a direct and significant effect on learning outcomes.

Interaction as an external supportive behavior from the teacher or classmates positively affects learning outcomes (She et al., 2021). Effective interactive content, which reduces network loss and promotes high levels of learner engagement in learning activities, has a positive impact on learning outcomes (Kim and Kim, 2021). Teacher-student interaction is a key aspect of the three types of interaction, also known as social interaction (Tuovinen, 2000). It is directly related to guided feedback and personalized support for teaching and learning, and is associated with learner satisfaction, and learning outcomes (Molinillo et al., 2018). When teachers lack experience in using GenAI to support learning or organize collaborative activities, learners feel less engaged, less motivated (Kuo et al., 2014). Learning can be significantly enhanced once supportive teacher interactions are increased and a more dynamic and responsive learning environment is created (Pahi et al., 2024). When conducting self-directed learning, learning peers’ interactions mainly take place through topic discussions and dialogs, and effective interactions can improve learning outcomes and develop higher-order thinking skills (Lee and Choi, 2017).

*H4*: Teacher-student interaction has a direct and significant effect on student-student interaction.

*H5*: Teacher-student interaction has a direct and significant effect on learning outcomes.

*H6*: Student-student interaction has a direct and significant effect on learning outcomes.

There are differences in the needs of students in different majors for GenAI, with art students preferring GenAI to stimulate creativity and provide a variety of outputs, while technology students are more concerned with accuracy and efficiency (Kim et al., 2025b). Teachers are the guides and organizers of teaching and learning activities, and their attitudes toward GenAI-supported learning, their ability to organize teaching and learning activities are important factors for effective teacher-student interaction. Learning activities with higher interactivity will increase motivation, improve learning outcomes and satisfaction compared to less interactive learning environments (Liaw and Huang, 2013). Learners’ achievement of satisfactory learning outcomes is an important external condition, which has a significant effect on learners’ learning motivation and learning satisfaction (Wu et al., 2010).

*H7*: Learning environment has a direct and significant effect on learning satisfaction.

*H8*: Teacher-student interaction has a direct and significant effect on learning satisfaction.

*H9*: Learning outcomes has a direct and significant effect on learning satisfaction.

**Figure 1:**
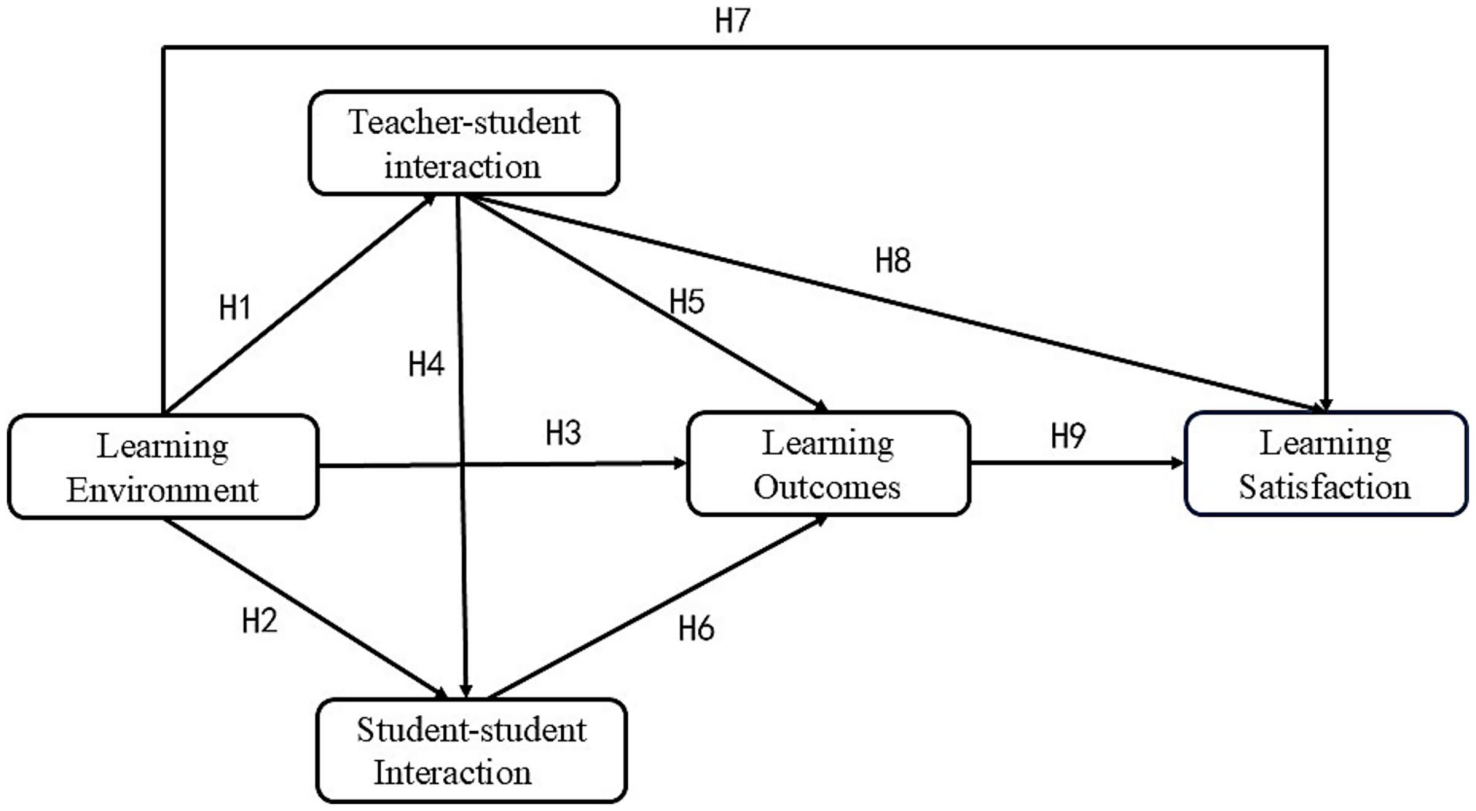
Research hypothesis model diagram.

Based on the above assumptions, we constructed a GenAI-supported hypothetical model of factors influencing college students’ MOOCs learning satisfaction from the perspective of learning experience, as shown in Figure 1. In order to simplify the variable names, we abbreviate learning environment as LE, teacher-student interaction as TSI, student–student interaction as SSI, learning outcome as LO, and learning satisfaction as LS in the subsequent articles.

## 4 Methodology

### 4.1 Research area and subjects

#### 4.1.1 Research area

In this study, we established a GenAI-supported MOOC learning environment within the national-level MOOC course “Modern Educational Technology” at Shaanxi Normal University. Offered to all students across the university, the course has undergone continuous updates since its launch in 2016. During its development and refinement, generative AI was integrated into key aspects including course design, instructional organization, learning support, and teaching evaluation, thereby achieving intelligent optimization throughout the entire MOOC learning process.

GenAI–Supported MOOCs Learning Environment refers to an intelligent learning ecosystem built by integrating generative AI technologies (such as DeepSeek, ChatGPT, etc.) into MOOCs course platforms (Cordero and Cordero-Castillo, 2025; Zawacki-Richter et al., 2019). Within this environment, MOOCs course platforms integrate with generative AI large models (e.g., DeepSeek, ChatGPT) to provide learners with intelligent content generation, interactive Q&A, and data feedback capabilities. This fosters a personalized learning space characterized by human-machine collaboration, enhancing the interactivity and adaptability of MOOCs education.

#### 4.1.2 Participants

To ensure the reliability and validity of the adapted research instrument, exploratory factor analysis (EFA) and confirmatory factor analysis (CFA) must be conducted on the questionnaire. EFA serves as a method for preliminary model exploration and construction based on a given sample, while CFA tests whether the theoretical model derived from confirmatory factors is robust and reproducible using a new sample. The samples used for these two analyses must be independent to ensure the identified factor structure is stable and generalizable. Therefore, we collected Samples A and B.

Sample A (questionnaire test data) is from Shaanxi Normal University, which, as a key university under the “211 Project,” is directly under the jurisdiction of the Ministry of Education of China, and its student population can represent the typical characteristics of Chinese college students to a certain extent. When conducting exploratory factor analysis, it is generally recommended that the sample size (N) be maintained at a ratio of 10:1 to 20:1 relative to the number of items (P) to stabilize the identification of latent dimensions within the questionnaire. A larger sample size yields more stable estimates of statistical indicators such as factor loadings and commonality, resulting in a more reliable factor structure (Costello and Osborne, 2005). Therefore, we selected approximately 300 college students who had taken the “Modern Educational Technology” course during the first semester of the 2024–2025 academic year through random sampling.

Sample B (official data from the questionnaire) was also from Shaanxi Normal University. This sample is independent from Sample A and is used for confirmatory factor analysis (CFA) and model building, also requiring adherence to sample size requirements and proportionality principles. However, CFA and structural equation modeling (SEM) necessitate estimating more parameters for complex model fit testing, typically demanding larger sample sizes (Kyriazos, 2018). To ensure the rationality and scientific validity of the sampling, this study employed stratified random sampling to select 402 students who had taken the “Modern Educational Technology” course during the first semester of the 2024–2025 academic year. Stratified random sampling is a scientific probability sampling method. It first divides the population into several mutually exclusive strata with similar internal characteristics based on specific criteria. The sample size for each stratum is then determined according to its proportion within the population. Samples are independently and randomly drawn from each stratum, ultimately combined to form the total sample (Cochran, 1977). The specific sampling steps are as follows: First, based on the enrollment categories for teacher education programs at Shaanxi Normal University, 12 major strata were selected as the primary stratification units, covering students with diverse academic backgrounds such as liberal arts (politics, English) and science/engineering (physics, mathematics, computer science). Within each major stratum, a second-level stratification was conducted based on academic performance in major courses, dividing students into three academic achievement strata. This ensured that each academic achievement stratum contained at least 10 samples, and the total sample size for each major stratum was no less than 30. Additionally, sampling considers gender ratios to prevent gender-based sample sizes from affecting the generalizability of results. All participants in Sample B completed the GenAI-supported MOOC course Modern Educational Technology and fulfilled related learning tasks. Before the course concluded, we invited students to participate in an online electronic questionnaire survey via classroom announcements and online notifications. The demographic data and the sources of the research subjects’ majors are shown in Tables 2, 3, including 225 male students (56%) and 177 female students (44%). All of them used GenAI-supported learning to varying degrees in their daily studies, including self-directed exploration of GenAI-supported learning styles and GenAI-supported learning tasks assigned by their schools or teachers. Regression analyses were performed on demographic variables (gender, age and grade), and the R2 showed a small effect, so their effects on learning experience and learning satisfaction were not considered.

**Table 2:** Demographic data.

Sociodemographic	Gender		Age			
	Male	Female	18	19	20	21
Frequency	225	177	33	273	91	5
Percent (%)	56	44	8.2	67.9	22.6	1.2

**Table 3:** The professional sources of the research subjects.

Professional	Number	Proportion (%)
Ideological and political education	35	8.7
Preschool education	34	8.5
Chinese language and literature	38	9.5
History	39	9.7
English	33	8.2
Biological science	30	7.5
Computer science and technology	32	8
Psychology	33	8.2
Geographical science	33	8.2
Chemistry	32	8
Mathematics and applied mathematics	30	7.5
Physics	33	8.2

### 4.2 Research tools

#### 4.2.1 Questionnaire design and description of variables

The questionnaire was divided into three parts, the first part was a survey of basic information, including gender, age, grade level and use of GenAI-supported learning. The second part was a survey on GenAI-supported MOOC learning experience, which was a revision of the scale developed by (Paechter et al., 2010), with 14 questions, using a Likert scale of 1–6 (from 1 “Totally disagree” to 6 “Totally agree”) was used to assess the learning experience of college students in different dimensions (LE, TSI, SSI, LO). The third section, the Learning Satisfaction Survey, was an adaptation of the questionnaire developed by(S. J. Lee et al., 2011), which consisted of four questions on a 1-6-point Likert scale (ranging from 1 “Totally disagree” to 6 “Totally agree”) to assess student satisfaction with learning, the questionnaire is detailed in Appendix A.

#### 4.2.2 Data collection and analysis methods

The survey was conducted anonymously via an online questionnaire. We posted a link to the questionnaire near the end of the MOOC course, explaining the purpose of the study and emphasizing that the data would only be used for academic research. Students participated voluntarily and were rewarded with a small number of course usual points to increase the response rate. After cleaning and screening the questionnaire data, analysis was conducted using SPSS 25 and AMOS 26 software. First, exploratory factor analysis was performed on Sample A (pilot data) using SPSS 25 to determine the dimensions of the adapted learning experience questionnaire and to test the reliability and validity of the items. Subsequently, after conducting reliability and validity tests, descriptive statistics, and correlation analyses on Sample B (formal data), confirmatory factor analysis was performed using AMOS 26 to verify that the learning experience dimensions in the formal questionnaire aligned with the exploratory factor analysis results. Finally, structural equation modeling (SEM) was constructed using AMOS 26 to examine the overall model fit and specific path coefficients of the hypothesized model.

#### 4.2.3 Exploratory factor analysis

An exploratory factor analysis was conducted on the learning experience section of the 300 samples of the pretest sample A using SPSS 25, and it was found that the 14 topics of learning experience were categorized in 4 dimensions (LE, TSI, SSI, LO). The reliability of the questionnaire was checked and the results showed that the KMO value for the learning experience section was 0.875 > 0.8, *p* < 0.001, and for the four topics of satisfaction the KMO = 0.774, alpha = 0.786, and the results indicated that the questionnaire reliability was acceptable.

The results of exploratory factor analysis showed that the eigenvalues and variances of the four components were 1.597 and 66.65%, respectively. Further EFA analysis using the rotated component matrix showed that all factor loadings of the study variables were greater than the recommended threshold of 0.50, with factor loadings ranging from 0.637 to 0.847, indicating that the items used to measure the various constructs were acceptable (Table 4).

**Table 4:** EFA via the rotated component matrix.

Variables	Code	Component			
		1	2	3	4
Teacher-student interaction (TSI)	TSI3	0.800			
	TSI4	0.755			
	TSI2	0.717			
	TSI1	0.651			
Learning outcomes (LO)	LO4		0.800		
	LO3		0.746		
	LO2		0.710		
	LO1		0.700		
Student–student interaction (SSI)	SSI2			0.847	
	SSI3			0.840	
	SSI1			0.827	
Learning Environment (LE)	LE3				0.740
	LE2				0.738
	LE1				0.637

#### 4.2.4 Confirmatory factor analysis

After determining the structure of the relationship between the variables and the adequacy and validity of the dataset through EFA, a validation factor analysis was also conducted using AMOS software. Data reliability was assessed using Cronbach’s alpha (*α*) and composite reliability, which was indicated as acceptable at *α* > 0.70. The internal consistency of the questionnaire was checked and the results showed significant CFA loadings for all variables (*p* < 0.001, *α* > 0.7). AVE and CR values were used to discriminate the validity of the dataset, and all variables satisfied AVE > 0.5 and CR > 0.7 except LE. however, when 0.4 < AVE < 0.5 and α > 0.6, the results were acceptable as shown in Table 5. The correlation coefficients are shown in Table 6, with diagonal lines showing the AVE values for each variable.

**Table 5:** CFA result reliability and validity.

Items	Estimate	S. E.	*p*	*α*	CR	AVE
LE1 < --LE	0.687			0.708	0.727	0.472
LE2 < --LE	0.751	0.084	***			
LE3 < --LE	0.616	0.103	***			
TSI1 < --TSI	0.753			0.845	0.850	0.587
TSI2 < --TSI	0.723	0.052	***			
TSI3 < --TSI	0.801	0.060	***			
TSI4 < --TSI	0.784	0.058	***			
SSI1 < --SSI	0.783			0.868	0.871	0.692
SSI2 < --SSI	0.869	0.068	***			
SSI3 < --SSI	0.842	0.061	***			
LO1 < --LO	0.589			0.797	0.808	0.517
LO2 < --LO	0.639	0.086	***			
LO3 < --LO	0.830	0.097	***			
LO4 < --LO	0.790	0.095	***			

1***, significant correlation at the 0.01 level (two-tailed).

**Table 6:** Correlation and discriminant validity results.

Variables	LE	TSI	SSI	LO
LE	0.472			
TSI	0.487^***^	0.587		
SSI	0.506^***^	0.618^***^	0.692	
LO	0.570^***^	0.563^***^	0.505^***^	0.517
Square root of AVE	0.687	0.766	0.832	0.719

1 ***, significant correlation at the 0.01 level (two-tailed).

**Figure 2:**
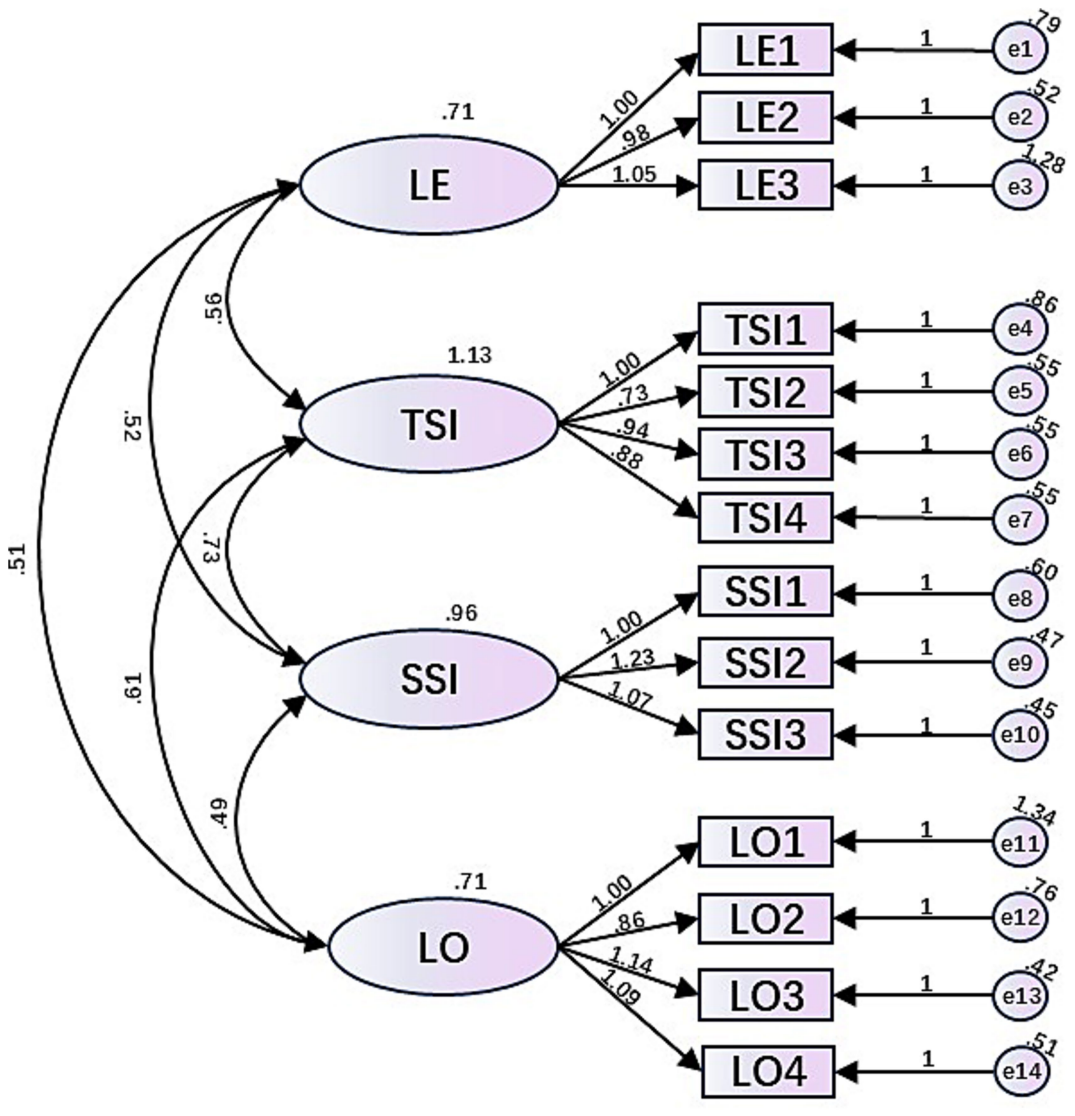
Confirmatory factor analysis.

During the CFA process, model fit indices such as sample size dependent chi-square (*χ*^2^), standardized root means square residuals (SRMR<0.06), relative chi-square indices (*χ*^2^/df < 5), comparative fit indices (CFI > 0.90), root mean square error of approximation (RMSEA<0.08) and Bentler-Bonett fit indices (NFI > 0.90) for acceptability. The structure produced model fit indices of chi-square (CMIN) = 255.186, degrees of freedom (df) = 71, relative chi-square index (CMIN/df) = 3.031, comparative fit index (CFI) = 0.947, standardized root-mean-square residuals (SRMR) = 0.0447, and root-mean-square error of approximation (RMSEA) = 0.071. The results indicate that the model fit of the study is good and suitable for further analysis (Figure 2). In conclusion, the questionnaire has high reliability and validity and can be used for subsequent model testing.

## 5 Results

### 5.1 Status of learning satisfaction of college student in GenAI-supported MOOC learning

The Mean and Standard Deviation of learning satisfaction were calculated to understand the current status of GenAI-supported MOOC learning satisfaction among college students. The results show that LS (*M* = 4.257, SD = 1.233) indicates that college students have high overall satisfaction with the GenAI-supported MOOC courses they participate in, which is in high consistency with the results of previous studies. MOOC is not constrained by time and place, and provides a practical path for educational equity and personalized education. College students have diverse learning needs, and learning through MOOCs can meet their learning needs with personalized learning experience with high satisfaction (Aznar-Díaz et al., 2024; Le, 2025).

### 5.2 Correlation analysis of GenAI-supported MOOC learning experience and learning satisfaction

The relationship between GenAI-supported MOOC learning experience and learning satisfaction (LS) was examined using Pearson matrix correlation analysis (r) (Table 7), which showed that LS was associated with LE (*r* = 0.515, *p* = 0.01), LS with TSI (*r* = 0.460, p = 0.01), LS with SSI (*r* = 0.443, *p* = 0.01), LS with LO (*r* = 0.730, *p* = 0.01). This suggests that once the quality of the learning environment improves, teacher-student interaction, and student–student interaction improves, students obtain better learning outcomes and subsequently have better learning satisfaction. In this case, learning satisfaction LS (*M* = 4.257, SD = 1.233), indicates that the overall student satisfaction is high in the process of using GenAI tools to support learning. The lowest scores in the dimensions of learning experience were for learning outcomes LO (*M* = 4.517, SD = 0.970), SSI (*M* = 4.652, SD = 1.152), TSI (*M* = 4.759, SD = 1.025), and LE (*M* = 4.798, SD = 1.002).

**Table 7:** Correlation and discriminant validity results.

Variables	LE	TSI	SSI	LO	LS
LE	1.000				
TSI	0.487^***^	1.000			
SSI	0.506^***^	0.618^***^	1.000		
LO	0.570^***^	0.563^***^	0.505^***^	1.000	
LS	0.515^***^	0.460^***^	0.443^***^	0.730^***^	1.000
Mean	4.798	4.759	4.652	4.517	4.257
Std. Deviation	1.002	1.025	1.152	0.970	1.233

1 ***, significant correlation at the 0.01 level (two-tailed).

### 5.3 Testing and modification of the impact factor model

**Figure 3:**
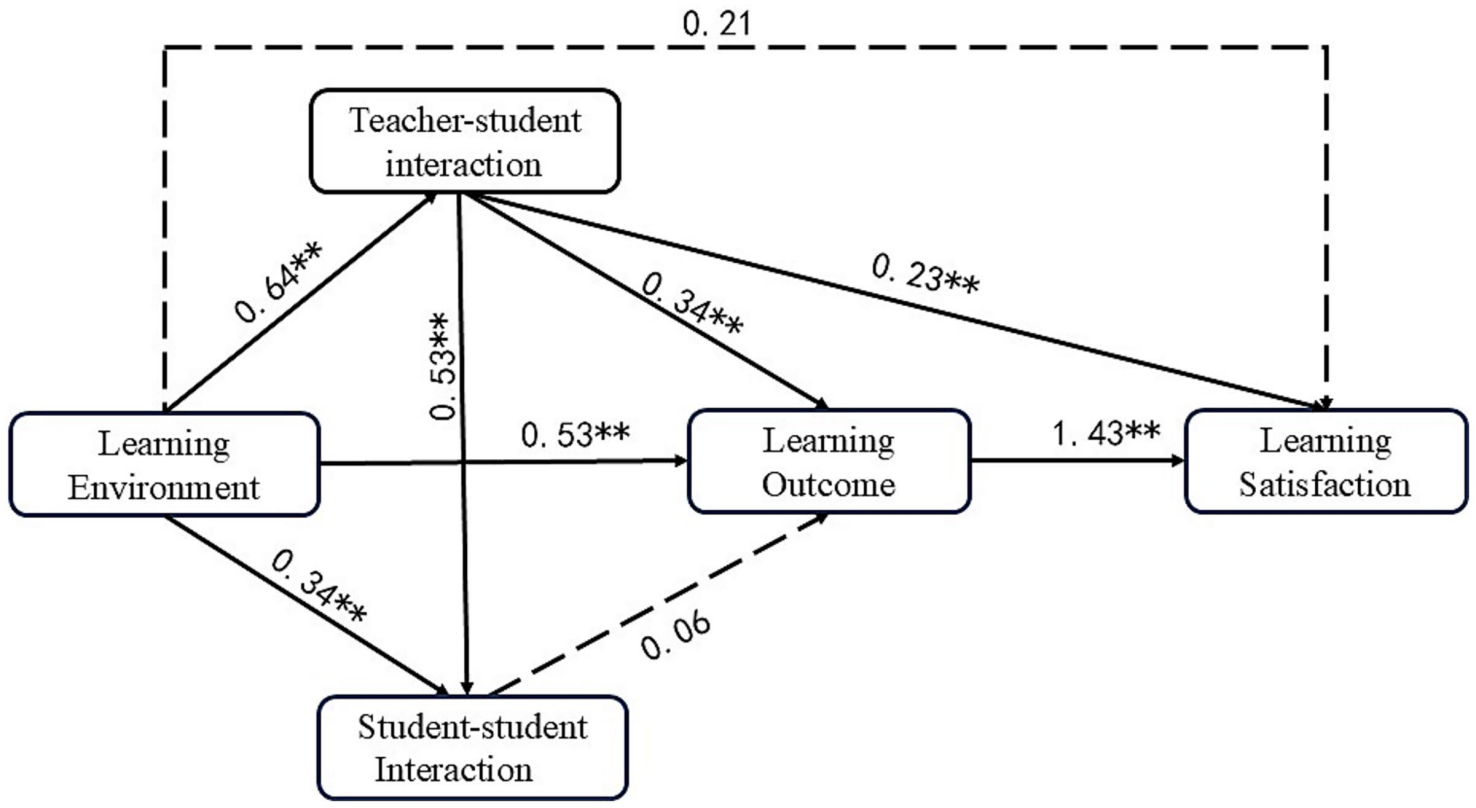
Model fitting results.

The effects of LE, TSI, SSI, and LO on LS were examined using SEM, and a model of university students’ MOOC learning satisfaction supported by GenAI under the perspective of learning experience was constructed. Based on the results of the initial model, e13-e14 and e17-e18 were corrected. The structural model of the main effect after correction is shown in Figure 3, which has a good fitting effect. Among them, CMIN = 322.999, df = 124, CMIN/df = 2.605, CFI = 0.949, GFI = 0.920, AGFI = 0.890, NFI = 0.920, RFI = 0.901, IFI = 0.949, TLI = 0.937, SRMR = 0.0440, RMSEA = 0.063.

The results show (shown in Table 8) that H1, H2, H3, H4, H5, H8, and H9 are supported, i.e., LE has a significant effect on TSI, SSI, and LO, whereas the direct effect of the LE on LS is not significant, i.e., H7 is not significant; the effect of TSI on SSI and LO is significant, and TSI on LS has a negative effect is significant; the effect of SSI on LO is not significant, i.e., H6 is not significant.

**Table 8:** The relationship between GenAI-supported online learning experiences and satisfaction.

Hypotheses	Path	Estimate	S. E.	C. R.	*p*	Label
H1	LE--- > TSI	0.622	0.076	8.439	***	Significant
H2	LE --- > SSI	0.315	0.078	4.351	***	Significant
H3	LE --- > LO	0.516	0.094	5.709	***	Significant
H4	TSI--- > SSI	0.506	0.073	7.210	***	Significant
H5	TSI--- > LO	0.335	0.08	4.183	***	Significant
H6	SSI--- > LO	0.063	0.065	0.931	0.352	Insignificant
H7	LE --- > LS	−0.167	0.141	−1.467	0.142	Insignificant
H8	TSI--- > LS	−0.197	0.101	−2.332	0.020	Significant
H9	LO--- > LS	1.199	0.203	7.076	***	Significant

1 ***, significant correlation at the 0.01 level (two-tailed).

### 5.4 Establish a theoretical model of influencing factors

**Figure 4:**
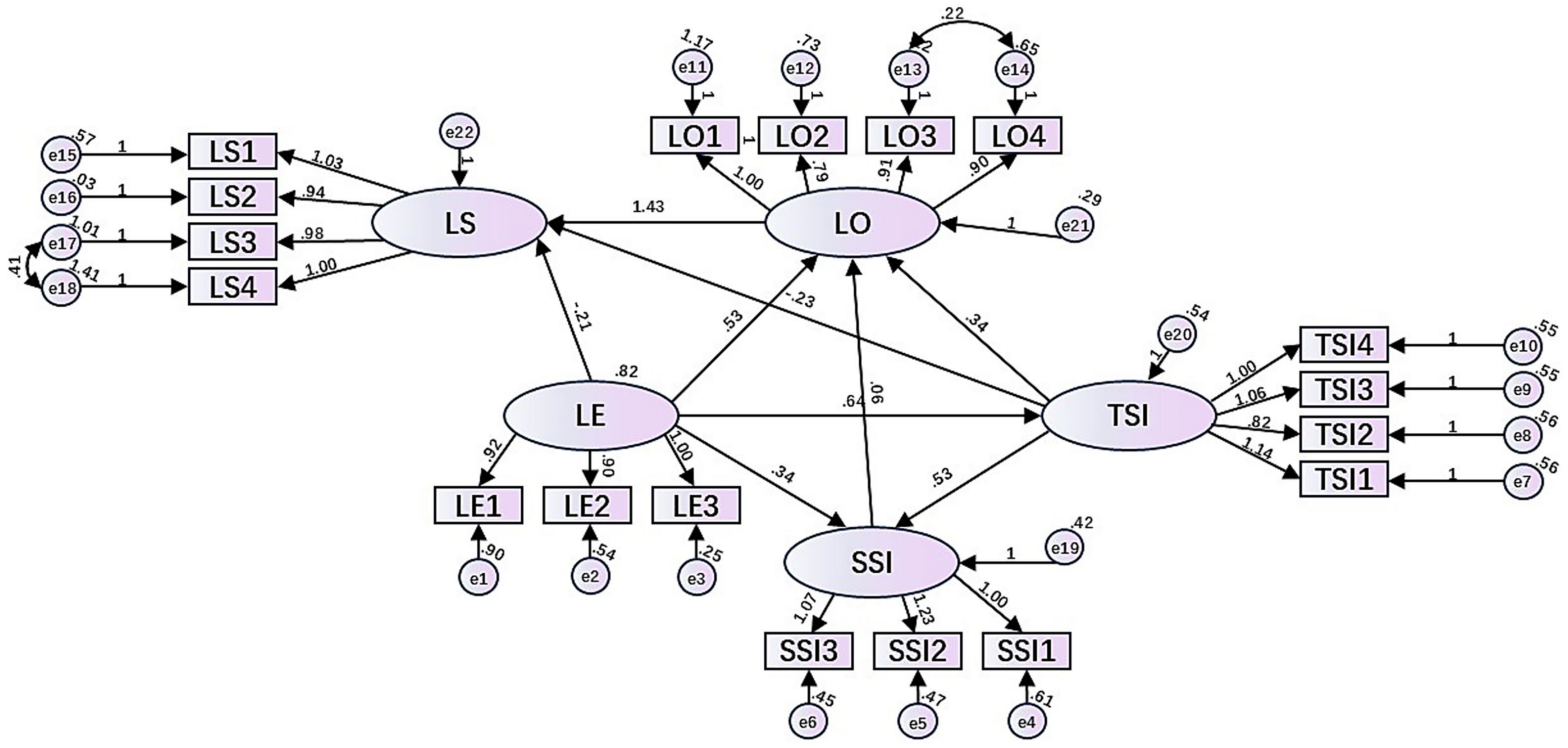
The GenAI-supported MOOC learning satisfaction model for college students from the perspective of learning experience.

Based on the results of the above path analysis, we constructed a model of factors influencing college students’ learning satisfaction in the GenAI-supported MOOC learning environment from the perspective of learning experience, as shown in Figure 4. The model shows that when college students use GenAI-supported MOOC learning, LE has a significant positive effect on TSI, SSI and LO, but has no significant effect on LS; TSI has a significant positive effect on SSI and LO, but has a negative and significant effect on LS; SSI has a non-significant effect on LO, and LO have a significant direct effect on LS.

## 6 Discussion

This study explores the factors influencing college students’ learning satisfaction in a GenAI-supported MOOCs environment and verifies most of the theoretical hypotheses. On this basis, we discuss the key results.

### 6.1 Discussion on the current state of satisfaction

Research findings indicate that college students exhibit overall high learning satisfaction (*M* = 4.257) in GenAI-supported MOOC learning environments. This result aligns with previous related studies (Kumar and Kumar, 2020). As an open online course format, MOOCs offer high flexibility and personalized features, enabling students to achieve positive learning experiences. In this study, university students demonstrated generally high satisfaction with GenAI-supported MOOC courses. This indicates that the introduction of GenAI technology did not negatively impact learner satisfaction; rather, it further enhanced student satisfaction through timely Q&A support and personalized guidance. Indeed, numerous studies have examined the impact of GenAI tools on learning satisfaction in educational settings. Integrating GenAI technology into MOOCs provides learners with richer forms of support and interaction, aligning with prior research findings that GenAI enhances student satisfaction (Baig and Yadegaridehkordi, 2025; Li and Chiu, 2025). Overall, learners demonstrated high satisfaction with GenAI-supported MOOC courses, indicating widespread recognition of the value of this novel learning environment. This finding also signals that effectively leveraging GenAI technology can maintain or even enhance learner satisfaction. Of course, we must acknowledge potential individual differences underlying this high satisfaction—some learners may have rated the experience highly due to the novelty of GenAI (Wu et al., 2025).

### 6.2 Discussion on factors influencing learning satisfaction

#### 6.2.1 GenAI-supported MOOC learning environment

Research findings indicate that GenAI-supported MOOC learning environments exert a significant positive influence on TSI, SSI, and LO, but do not significantly enhance LS. First, LE positively promotes TSI, consistent with previous research findings (Miao et al., 2022). A well-designed online learning environment (e.g., a fully functional learning platform, abundant course resources) facilitates teacher-student interaction (Xiao et al., 2023). When the technological environment facilitates communication, TSI become easier to initiate and enhance in quality. Particularly within MOOC learning environments, GenAI can play multiple roles to strengthen TSI. For instance, it can serve as a “virtual study companion,” provide personalized Q&A support, offer feedback and assistance to promote collaborative and collective learning, and significantly facilitate discussions among learning peers (Huang, 2024; Lu and Ba, 2025). Thus, LE actively supported by GenAI foster efficient TSI. Future practices should further refine online teaching platforms and GenAI support functions to provide smoother communication environments and richer interactive formats, fully leveraging the technological environment’s role in facilitating teacher-student exchanges.

Secondly, GenAI-supported learning environment experience also positively impacts SSI. This finding indicates that when equipped with a conducive learning environment, students are more likely to engage in collaboration and communication. Particularly within MOOCs learning environments, the motivation and quality of SSI largely depend on the collaborative tools provided by the platform (Martín-Monje et al., 2018). Research indicates that GenAI can generate discussion topics based on course content or simulate dialog to participate in group discussions, thereby helping learners cultivate a positive seminar atmosphere and enhancing SSI (Lee et al., 2024). Consequently, future practices should prioritize leveraging GenAI to strengthen SSI. For instance, utilizing GenAI’s personalized support can promote online collaborative learning among learners, enhancing both the convenience and depth of peer communication.

Furthermore, GenAI-supported learning environment experience has a significant positive impact on LO. This conclusion aligns with previous research on MOOC platforms: user-friendly learning platforms, appropriately designed courses, and well-structured activities all facilitate sustained student engagement, thereby reducing dropout rates and yielding better LO (Choi et al., 2014; Molinillo et al., 2018). When MOOC platforms are user-friendly, students can focus more intently on the learning content itself, minimizing distractions caused by technical issues. Simultaneously, well-designed courses maintain student motivation and enhance learning effectiveness. For educators, this implies that MOOC design should prioritize creating high-quality technical environments and course content to maximize student learning outcomes.

It is noteworthy that this study found that the GenAI-supported learning environment experience did not have a direct significant impact on LS. In other words, simply improving the quality of the learning environment does not directly translate into higher student satisfaction with the course. The reason behind this result may lie in the fact that the learning environment’s effect on LS is mediated indirectly through influencing other intermediate variables. According to the stimulus-organism-response (S-O-R) model in environmental psychology, environmental factors act as external stimuli that first influence an individual’s internal state before affecting behavioral responses (Kawaf, 2012). In this study’s context, the GenAI learning environment may indirectly influence LS by first enhancing students’ interaction quality and LO. This partially explains why the learning environment pathway to satisfaction was not significant in this study. The benefits of the environment may have already manifested in higher interaction quality and learning outcomes, with satisfaction being directly influenced by these factors. This aligns with previous research findings that environments affect LS by influencing learners’ perceived usefulness and learning effectiveness (Gray and Diloreto, 2016). Therefore, while the learning environment does not directly affect student learning satisfaction, this does not imply its irrelevance. Rather, it reminds educators to focus on the pathways through which environmental factors influence learners’ experiences and outcomes, thereby shaping satisfaction.

#### 6.2.2 Teacher-student interaction

This study found that TSI significantly and positively influenced both SSI and LO, yet it significantly and negatively impacted LS. These findings warrant further discussion. Specifically, first, TSI significantly enhanced SSI, consistent with prior research: when designing instructional activities, teachers’ choices regarding interaction methods and content significantly affect the quality of SSI. When guiding students in using GenAI to support learning, teachers’ suggestions for grouping and discussion facilitation can optimize SSI processes, enhance collaborative efficiency, and thereby promote peer collaboration (An et al., 2025). Effective teacher intervention can stimulate more positive interactions among students. In future practice, teachers should further leverage the functional characteristics of GenAI and integrate it into MOOC courses. For instance, GenAI-generated content can be used as discussion material for online learners, and students can be guided to collectively evaluate GenAI responses, thereby deepening and broadening peer-to-peer assistance.

Second, SSI has a significant positive impact on LO, consistent with extensive research findings (Kamran et al., 2023; Lin et al., 2020; Offir et al., 2003). Teacher guidance and feedback remain critical factors influencing student academic performance (Gan et al., 2021). While GenAI can provide some level of question-answering and guidance, the role of teachers remains indispensable. Abedi et al. note that teacher feedback ranks among the strongest factors influencing learning outcomes. In GenAI-supported learning, teachers can compensate for technological limitations by supplementing higher-order knowledge—such as correcting errors generated by GenAI—thereby enabling students to develop more robust knowledge structures and learning outcomes (Abedi et al., 2023). This further corroborates our finding that teacher-student interaction enhances learning effectiveness. In future practice, MOOC classrooms should actively integrate GenAI technology as an effective tool to facilitate SSI, thereby enhancing learners’ educational outcomes.

Finally, the study found that TSI had a negative impact on LS. Although this result was unexpected, it may have profound reasons in the context of GenAI-supported MOOC learning. First, the negative impact of TSI on learning satisfaction does not imply that interaction itself is detrimental. Rather, it reflects issues with the nature or quality of interaction within GenAI-supported MOOC learning environments. In such settings, GenAI can provide learners with timely, efficient, and comprehensive feedback (Wu et al., 2025). In contrast, particularly within MOOC settings, instructor guidance often exhibits delays and tends to focus primarily on knowledge transmission. This phenomenon may stem from instructors failing to effectively integrate GenAI-supported teaching activities, with their guidance remaining confined to traditional knowledge dissemination. Consequently, it falls short of meeting learners’ needs for autonomy and personalized feedback. Consequently, learners perceive a conflict between teacher guidance and GenAI support, diminishing their expectations for teacher-student interactions. This ultimately leads to TSI negatively impacting learning satisfaction (Yang Y. et al., 2025). Furthermore, the introduction of GenAI alters the form of traditional TSI; some students may prefer to autonomously use GenAI to find answers rather than engage frequently with teachers (Lu and Ba, 2025). Furthermore, emotional factors may influence the relationship between teacher-student interaction and learning satisfaction. Within this learning environment, learners are prone to negative emotions. While the novelty of GenAI and the sense of accomplishment upon achieving learning goals represent positive emotions, concerns about unfamiliar AI technology and frustration from improper use constitute negative emotions. Both types of emotions significantly impact learning satisfaction (Jin et al., 2025; Khlaif et al., 2024; Kim, 2023). In MOOC courses, teachers bear the responsibility of mitigating students’ negative emotions through motivation, empathy, and psychological support. However, if such support becomes perfunctory or duplicates GenAI feedback, students may perceive no added value and instead feel annoyed, negatively impacting LS (Yang et al., 2022). One learner candidly shared in a MOOC discussion forum: “I chose MOOCs to learn knowledge quietly at my own pace. If instructors assign excessive tasks or mandate frequent interactions, it makes me very resistant.” This illustrates how inappropriate or excessive teacher-student interaction can provoke dissatisfaction among some learners. In summary, the negative impact of TSI on satisfaction suggests that in GenAI-supported MOOC learning environments, instructors must redefine their roles. They should integrate GenAI’s rapid feedback with humanistic care to provide more effective student support (Yang H. et al., 2025). Future research should further explore how to optimize teaching interaction methods within GenAI-supported environments (Zhu et al., 2025), thereby addressing the detrimental effects of TSI on LS.

#### 6.2.3 Student–student interaction

The findings of this study reveal that SSI does not significantly impact LO, contrary to our initial expectations. Generally, SSI is believed to promote deeper understanding through discussion and knowledge sharing, thereby enhancing LO (Sein-Echaluce et al., 2016). However, in the context of GenAI-supported MOOCs, the failure of peer interaction to significantly improve LO may stem from multiple factors. First, the form and quality of SSI within MOOC environments are inadequate. MOOC learners are numerous and possess diverse educational backgrounds. Interactions among learners are often constrained by their professional backgrounds and foundational knowledge. When learners at different levels exhibit excessive knowledge disparities, interactions may become inefficient, rendering SSI largely superficial (Johnson, 1981). Second, GenAI has partially replaced certain aspects of SSI. When encountering difficulties, learners may prefer interacting with GenAI over engaging with peers. This scenario diminishes the impact of SSI, making LO more dependent on learner-GenAI interactions rather than peer collaboration. These findings suggest that within GenAI-supported MOOC learning environments, we must re-evaluate and redesign the role and methods of SSI, exploring the combined effects of different collaborative interaction combinations on LO (Bossér and Lindahl, 2019).

#### 6.2.4 Learning outcome

LO exert a significant positive influence on LS. This finding aligns with existing research: when students achieve expected LO, they demonstrate higher satisfaction with the learning process (Goh et al., 2017). In GenAI-supported MOOC learning environments, when learners enhance their LO through GenAI tools, their satisfaction with course arrangements and the tools used also increases accordingly. Conversely, if LO are unsatisfactory, their satisfaction decreases. This result highlights the significant impact of LO on learning satisfaction. It suggests that while we focus on improving TSI and LE experience, we should place greater emphasis on helping students achieve their expected learning goals. The same principle applies to GenAI applications: their value should extend beyond providing interaction and feedback to tangibly advancing students’ knowledge acquisition and skill development. Future research could further explore how different types of LO influence satisfaction levels, thereby addressing student satisfaction enhancement from multiple dimensions.

Overall, the contributions of this study fall into two categories: theoretical and practical. The theoretical significance is primarily reflected in the following aspects: existing research has largely focused on surveys and improvements of learning satisfaction within traditional MOOC learning environments, while there has been insufficient discussion regarding learning satisfaction in environments incorporating GenAI. Addressing this gap, this study systematically examines the factors influencing learning satisfaction within GenAI-supported MOOC environments. Our findings validate the applicability of established satisfaction theories in novel pedagogical contexts—for instance, the Stimulus-Organism-Response (S-O-R) model remains effective in GenAI-enabled learning settings (Kawaf, 2012). Simultaneously, we uncover new evidence, such as the negative impact of teacher-student interactions on satisfaction, providing a foundation for advancing satisfaction theory. Overall, this study fills the gap in understanding the mechanisms influencing learning satisfaction within GenAI-supported environments, enriching theoretical interpretations of learning satisfaction. Its practical implications are as follows: First, it validates the positive impact of GenAI-supported MOOC learning environments on student learning outcomes, thereby fostering learning satisfaction—a finding that positively influences future GenAI integration into broader educational contexts. Only through meticulous design and planning of GenAI applications within MOOC learning environments can its potential be fully realized. We hope these findings will assist higher education administrators and instructors in leveraging GenAI technology more effectively within MOOC settings, thereby creating superior learning experiences for university students.

## 7 Conclusion

From learning experience perspective, this study explored the influencing factors of college students’ learning satisfaction in GenAI-supported MOOC environments, and empirically tested the constructed hypothesized models to obtain a series of meaningful conclusions. It was found that in GenAI-supported MOOC learning, (1) the learning environment did not have a direct impact on learning satisfaction, but had a positive impact on satisfaction through learning outcomes; (2) teacher-student interaction had a positive impact on learning outcomes but a negative impact on satisfaction; and (3) student–student interaction, although they could enhance learning outcomes, did not have a significant impact on satisfaction. The results of this study point out the direction for subsequent development of teacher-student interaction and student–student interaction design in GenAI-supported MOOC learning. When designing interactive activities, teachers should pay more attention to the aspects that GenAI cannot replace, clarify the teacher’s role, and provide emotional support for learners. At the same time, they should balance the roles of humans and computers, and design rationally assigned human-computer collaborative tasks to enhance the effectiveness of student–student interactions. In addition, the ethical issues brought by GenAI should not be ignored. When carrying out GenAI-supported learning activities, teachers should establish an appropriate learning evaluation system to ensure the effectiveness of the learning activities.

Compared with other related studies, we start from the perspective of learning experience, and this study constructs a model of online learning satisfaction that incorporates technology-enabled factors, enriching the theoretical framework in the field of online education. While previous studies have mostly drawn on the customer satisfaction model to explore online learning satisfaction (Anderson and Sullivan, 1993), this study takes generative AI factors into account, expanding the connotation of learning experience theory and confirming the important role of variables such as teacher-student interaction and learning outcomes, which provides a reference paradigm for subsequent studies to further explore learning satisfaction in other online learning environments.

## 8 Limitations and future research

Although this study has made some progress in exploring the factors influencing learning satisfaction in GenAI-supported MOOC learning environments, its findings must be interpreted within the context of several limitations. First, limitations exist in sample selection. To maximize representativeness and randomness, we employed stratified random sampling; however, selection bias was difficult to completely avoid during implementation. For instance, the surveyed learners may have been above average in overall proficiency, potentially influencing the findings. Future research should broaden the sample scope to include more diverse learner groups, enhancing representativeness. Second, the generalizability of the findings is limited. The sample primarily consists of Chinese university students, whose learning habits and knowledge foundations may differ significantly from those of students in other countries and regions. Therefore, future research should conduct cross-cultural comparisons, incorporating data from learners across different nations, educational systems, and linguistic backgrounds to test the applicability of the learning satisfaction influence model in multicultural settings. Third, this study employs a cross-sectional design, with data collection concentrated within a short time window. Although we endeavored to control for temporal interference, students’ perceptions of learning satisfaction, learning environment, teacher-student interaction, and peer-student interaction may change over time (Sidik and Syafar, 2020). Therefore, future research could employ longitudinal studies or mixed-method approaches, utilizing multi-stage data collection to elucidate the evolution of learner satisfaction and its multidimensional determinants (Bartolucci, 1980). Fourth, this study primarily relies on self-reported data. While this method holds significant value across multiple research domains, it carries inherent risks such as recall bias and distortions in subjective perceptions (Fryer and Dinsmore, 2020). To ensure research accuracy, subsequent studies should incorporate qualitative data such as learning process records and interview transcripts to achieve triangulation. Overall, while this study has the aforementioned limitations, these shortcomings point the way forward for future research. Future studies are expected to delve deeper into the influence mechanisms of learning satisfaction within GenAI-supported MOOC learning environments, thereby advancing MOOC optimization and enhancing learner outcomes.

## Data Availability

The raw data supporting the conclusions of this article will be made available by the authors, without undue reservation.
