# Supplementary material for: Exploring the factors influencing college students’ learning satisfaction in generative AI-supported MOOCs learning environment: a learning experience framework perspective
Source: Front Psychol. 2025 Oct 27;16:1633686. doi: 10.3389/fpsyg.2025.1633686 (PMC12597930; doi:10.3389/fpsyg.2025.1633686)
Supplement: Supplementary file 1 [file Supplementary_file_1.docx]

Supplementary Material

# Appendix A: Questionnaire on GenAI-supported MOOC Learning Experience and Learning Satisfaction for College Students

GenAI-supported MOOC Learning Experience:

| **Items** | **Totally disagree** | **Disagree** | **Somewhat disagree** | **Somewhat agree** | **Agree** | **Totally agree** |
| --- | --- | --- | --- | --- | --- | --- |
| 1.In GenAI-supported MOOC learning, the learning environment can provide chats, thematic tasks, and other means of accessing learning materials to achieve learning objectives. | 1 | 2 | 3 | 4 | 5 | 6 |
| 2.GenAI-supported MOOC learning in which GenAI can provide logical and well-organized learning materials. | 1 | 2 | 3 | 4 | 5 | 6 |
| 3.GenAI-supported MOOC learning is easy to operate and conduct. | 1 | 2 | 3 | 4 | 5 | 6 |
| 4.I was able to easily get in touch with the instructor when I needed advice/guidance on GenAI-supported MOOC learning. | 1 | 2 | 3 | 4 | 5 | 6 |
| 5.Teachers have a high level of expertise in conducting GenAI-supported MOOC learning. | 1 | 2 | 3 | 4 | 5 | 6 |
| 6.My teacher was able to answer questions related to my participation in the GenAI-supported MOOC learning in a variety of ways, including lectures and demonstrations. | 1 | 2 | 3 | 4 | 5 | 6 |
| 7.My teacher was able to support and advise me on my participation in GenAI-supported MOOC learning. | 1 | 2 | 3 | 4 | 5 | 6 |
| 8.I can quickly exchange knowledge and skills, etc. with other students participating in GenAI-supported MOOC learning via email, chat, and threaded exchanges. | 1 | 2 | 3 | 4 | 5 | 6 |
| 9.I had a lot of problems with communicating with other students in the GenAI-supported MOOC study. | 1 | 2 | 3 | 4 | 5 | 6 |
| 10.Participating in GenAI-supported MOOC learning facilitates me to communicate with other students together. | 1 | 2 | 3 | 4 | 5 | 6 |
| 11.GenAI-supported MOOC learning in which the GenAI environment can provide ways to detect the growth of my knowledge (e.g., generating test questions, etc.). | 1 | 2 | 3 | 4 | 5 | 6 |
| 12.Participating in GenAI-supported MOOC learning, I gained (conceptual) knowledge of the subject matter. | 1 | 2 | 3 | 4 | 5 | 6 |
| 13.Participating in GenAI-supported MOOC learning, I learned to apply my knowledge to solve different problems. | 1 | 2 | 3 | 4 | 5 | 6 |
| 14.Participating in a GenAI-supported MOOC, I acquired skills related to self-regulation . | 1 | 2 | 3 | 4 | 5 | 6 |

GenAI-supported MOOC Learning Satisfaction:

| **Items** | **Totally disagree** | **Disagree** | **Somewhat disagree** | **Somewhat agree** | **Agree** | **Totally agree** |
| --- | --- | --- | --- | --- | --- | --- |
| 1. Participation in GenAI-supported MOOC learning has enhanced my interest in learning about my subject. | 1 | 2 | 3 | 4 | 5 | 6 |
| 2.I feel that I met my learning objectives while participating in a GenAI-supported MOOC. | 1 | 2 | 3 | 4 | 5 | 6 |
| 3.I like the format of GenAI-supported MOOC learning. | 1 | 2 | 3 | 4 | 5 | 6 |
| 4.I would like to recommend GenAI-supported MOOC learning as a way to others. | 1 | 2 | 3 | 4 | 5 | 6 |
